# Sharing H5N1 Viruses to Stop a Global Influenza Pandemic

**DOI:** 10.1371/journal.pmed.0040330

**Published:** 2007-11-20

**Authors:** Laurie Garrett, David P Fidler

## Abstract

Indonesia's refusal to share samples of H5N1 virus with World Health Organization for most of 2007 is distressing and potentially dangerous for global public health, argue the authors.

Although the threat of pandemic influenza, spawned by continuing avian influenza A (H5N1) epidemics, has dropped off the front pages, concern among experts continues to grow. At the end of 2005, only 17 countries had H5N1 outbreaks in chickens, ducks, or humans [1]. As of September 2007, the virus has circulated in 60 countries [1], mutations have been reported (for example, in a patient in Turkey [2] and another in Thailand [3]), and virologists and public health officials nervously watch clusters of probable human-to-human spread of the virus, such as in Thailand in 2004 and Indonesia in 2006 [4]. For reasons not fully understood, most human cases and clusters of probable human-to-human transmission of H5N1 since January 2006 have occurred in Indonesia [5,6].

Recent studies have begun to characterize the mutations in H5N1 that may be a prerequisite for efficient human-to-human transmission [7,8]. The world needs to monitor each new influenza virus in order to check for such mutations, which could transform H5N1 into a dangerous pathogen easily spread between people. How devastating might such a transformation be? In an age of globalization and commercial air travel, estimating how great a toll a lethal human-to-human influenza virus could inflict is difficult. Estimates of deaths from the last great bird-to-human flu pandemic of 1918 range from 50 to 100 million [9], which provides a glimpse of the global damage that could be caused by a pandemic influenza accelerated by 21st century globalization.

## Indonesia's Refusal to Share Viruses

In light of the importance of virus monitoring for pandemic influenza preparedness and response, Indonesia's refusal to share samples of H5N1 virus with the World Health Organization (WHO) for most of 2007 is distressing and potentially dangerous for global public health. Negotiations with Indonesia to resume rapid and open virus sharing have proved difficult, with Indonesia repeatedly refusing to share unless significant changes were made to allow it greater access to vaccine derived from samples it shared with WHO [10].

Even though Indonesia resumed some virus sharing with WHO in the second week of September 2007 [11], the fundamental dispute is not resolved. Indonesia's willingness to continue virus sharing may depend on the outcome of intergovernmental negotiations in Geneva in November 2007 [12]. At present, prospects for overcoming the central disagreements do not appear good. We would like to suggest a way to break the root causes of the impasse, by taking a novel strategic approach to pandemic control and bringing new partners to the pandemic action table.

To begin, we need to understand why Indonesia took this radical position, and why it has gained support from other developing nations, even within the Asian influenza region. For the last 50 years, global influenza governance has operated as follows: WHO collaborating laboratories annually analyze samples of new influenza viruses circulating primarily in Asia. A WHO committee then determines which strains appear most likely to affect human populations in the coming months, and manufacturers start producing vaccine for those strains [13].

Typically some 250–300 million vaccine doses are made each year, and most of those vaccinated are residents of developed countries [14]. This inequitable situation creates concern, especially with the prospect of pandemic influenza increasing. Thus, many political and health officials are scrambling to find a way to increase production and equitable distribution of vaccine. We are currently limited, however, in vaccine technology, pharmaceutical industry incentives, and credible dissemination strategies in many developing countries.

So, as Asian countries have grown anxious about H5N1, and witnessed up to 100% fatality rates in infected chickens [15] and up to 70% mortality rates in infected humans [16,17], some developing countries have challenged the traditional global influenza strategy by asking, “What's in it for us? We share virus samples, and pharmaceutical companies make vaccines from them that primarily benefit rich countries. Without better access to vaccine, why should we share virus samples?”

WHO has tried to find a reasonable answer to that question, offering assurance to Indonesia and its sympathizers that the status quo will change. Scientists are trying to invent new vaccines that can protect against a broader range of strains, making it possible to build stockpiles for future use. In addition, pharmaceutical companies are making traditional vaccines against the current avian forms of H5N1.

The deeper problem is, however, that current pharmaceutical strategies for pandemic control basically offer protection to a small number of developed countries. For the rest of the more than 5 billion human beings on the planet, technological solutions are scarce, if not nonexistent.

## Stockpiling Control Tools in Hong Kong

The world needs a strategy to overcome the virus sharing impasse and the underlying sources of this problem. Influenza is essentially an aquatic bird virus naturally found among animals that migrate along the Asian flyway from Indonesia to Siberia, so targeted strategic stockpiling for that region makes epidemiological sense. We propose that annually updated supplies of more than 500 million doses of highly specific influenza vaccine, plus antiviral medicines, protective masks and gloves, and germicide washes be stockpiled in Hong Kong.

We select Hong Kong for three key reasons: It has demonstrated absolute transparency regarding disease emergences going back several decades, including the initial recognition of H5N1 in 1997. Hong Kong is also a dynamic center of virus research and response, pioneering most of what is now known about severe acute respiratory syndrome (SARS) and avian influenza. More importantly, Hong Kong sits in the middle of the ecological zone that has spawned the bulk of all influenza strains known to have emerged over the last three decades.

We advocate that the strategic stockpile be fed continuously and its specificity updated based on circulating forms of viruses. These objectives would be accomplished through an Advance Market Commitment (AMC) mechanism in which the G-8 nations and Asian powerhouses China, India, Singapore, South Korea, and Japan set aside a fund to guarantee purchase of stockpiled products.

The Asia-Pacific Economic Cooperation forum (APEC) should manage the AMC fund and the stockpiled materials in Hong Kong. APEC has proven to be one of the most dynamic and effective of the world's regional organizations. Through its Health Task Force and other activities [18], APEC leadership has recognized the need to forestall a devastating pandemic, and the APEC region already shoulders the burden of the ongoing H5N1 pandemic.

APEC has the financial and management capacity to oversee the AMC in a transparent and efficient manner, inviting donations from wealthy nations and philanthropies, as well as from its member states. APEC's political stature also gives it the authority to address the operational challenges created by the stockpile strategy, such as improving Asian developing countries' abilities to effectively distribute materials from the stockpile.

Finally, APEC has the diplomatic trust and political clout necessary to persuade Indonesia and other nations in Asia to share new viral samples with WHO on an urgent, timely, and consistent basis. APEC would rely upon WHO's technical advice in deciding when and how to use the stockpile, recognizing WHO's expertise in influenza virology and epidemiology, as well as in other emerging diseases.

This proposal raises questions about its epidemiological basis, implications for equitable vaccine distribution, and political feasibility. Epidemiologically, H5N1's spread beyond Asia perhaps increases the chances that the feared mutation might happen outside the APEC region. Although this is possible, our proposal relies on what most experts think is probable—that a pandemic strain is most likely to emerge from the Asian region. In addition, the APEC stockpile mechanism could set a precedent that other regional organizations could pursue.

Creation of the proposed stockpile might raise concerns that the AMC will exacerbate inequity for non-APEC developing countries by making stockpiled materials more scarce and costly. Again, this possibility cannot be dismissed lightly, but the AMC should increase global production capacities to fill the growth in demand, thus offering something existing approaches have failed to achieve—serious incentives for significant, sustainable increases in production capabilities. This mechanism can even work to encourage new capacity building in the Asian region.

## Breaking the Stalemate

In terms of the political feasibility of our proposal, we believe that the impasse over virus sharing, which threatens global health and international security, has to be addressed with new political strategies and innovative governance mechanisms. WHO and many countries view pandemic influenza as a security threat [19,20], revealing the importance of finding political solutions. Without new approaches to bridge the two sides of the dispute, prospects are grim and choices more drastic. Unresolved, this dispute could have two harmful consequences. First, it could threaten the improved cooperation against infectious diseases that has emerged in recent years, and splinter global health governance in ways that pit developed and developing countries against each other across a range of issues, from surveillance to intellectual property rights for pharmaceutical products.

Second, continued failure to break the stalemate may encourage countries threatened by the withholding of virus samples to pursue high-stakes strategies to break the deadlock, perhaps by seeking United Nations Security Council intervention on the grounds that failure to share viruses imperils global health security and international security. There is no way to predict the outcome of putting this issue before the Security Council.

The current stalemate poses such dangers that allowing the dispute to continue to drift and fester undermines prospects of finding solutions to the legitimate issues raised on both sides. An APEC-based strategic stockpile is one way to construct an epidemiologically valid and politically sensible path to ensuring that preparedness for pandemic influenza (and the next SARS or other emerging virus) does not disintegrate, leaving everyone at risk.

## Abbreviations

AMC: Advance Market Commitment
APEC: Asia-Pacific Economic Cooperation
SARS: severe acute respiratory syndrome
WHO: World Health Organization
